# Self-Consistent Field Modeling of Bottle-Brush with Aggrecan-like Side Chain

**DOI:** 10.3390/biomimetics10100694

**Published:** 2025-10-14

**Authors:** Ivan V. Mikhailov, Ivan V. Lukiev, Ekaterina B. Zhulina, Oleg V. Borisov

**Affiliations:** 1 NRC «Kurchatov Institute»—PNPI—IMC, St. Petersburg 199004, Russia; i.v.mikhailov-imc.ras@yandex.ru (I.V.M.); ivan.v.lukiev@gmail.com (I.V.L.); kzhulina@hotmail.com (E.B.Z.); 2 Center for Chemical Engineering, ITMO University, St. Petersburg 197101, Russia; 3 Institut des Sciences Analytiques et de Physico-Chimie pour l’Environnement et les Matériaux, UMR 5254 CNRS UPPA, 64053 Pau, France

**Keywords:** biomimetic bottle-brushes, aggrecan, double-comb polymers, self-consistent field theory

## Abstract

Bottle-brush polymers with aggrecan-like side chains represent a class of biomimetic macromolecules that replicate key structural and functional features of natural complexes of aggrecans with hyaluronic acid (HA) which are the major components of articular cartilage. In this study, we employ numerical self-consistent field (SCF) modeling combined with analytical theory to investigate the conformational properties of cylindrical molecular bottle-brushes composed of aggrecan-like double-comb side chains tethered to the main chain (the backbone of the bottle-brush). We demonstrate that the architecture of the brush-forming double-comb chains and, in particular, the distribution of polymer mass between the root and peripheral domains significantly influences the spatial distribution of primary side chain ends, leading to formation of a “dead” zone near the backbone of the bottle-brush and non-uniform density profiles. The axial stretching force imposed by grafted double-combs in the main chain, as well as normal force acting at the junction point between the bottle-brush backbone and the double-comb side chain are shown to depend strongly on the side-chain architecture. Furthermore, we analyze the induced bending rigidity and persistence length of the bottle-brush, revealing that while the overall scaling behavior follows established power laws, the internal structure can be finely tuned without altering the backbone stiffness. These theoretical findings provide valuable insights into relations between architecture and properties of bottle-brush-like supra-biomolecular structures, such as aggrecan-hyaluronan complexes.

## 1. Introduction

Aggrecans are the main structural components of articular cartilage, where they play a critical role in maintaining tissue hydration, resilience, and load-bearing capacity [1,2,3]. Large proteoglycans consist of a central core protein covalently attached to numerous side chains of glycosaminoglycan (GAG), primarily chondroitin sulfate (CS) and keratan sulfate (KS), that extend outward in a dense bottle-brush architecture. This strongly anionically charged structure generates a strong osmotic pressure, allowing the extracellular matrix to resist compressive forces and retain water, which is essential for the mechanical integrity and lubricating properties of cartilage [4]. The biological importance of aggrecans is underscored by their involvement in degenerative joint diseases such as osteoarthritis, where enzymatic degradation of aggrecan leads to loss of tissue function and progressive cartilage breakdown [5,6].

Synthetic bottle-brush polymers, also known as molecular brushes or cylindrical brushes, have been developed as biomimetic analogs of natural aggrecans [7,8]. These macromolecules feature a linear backbone densely grafted with polymeric side chains, leading to steric repulsion that promotes an extended conformation and results in a large hydrodynamic volume in solution, and to properties that closely resemble those of aggrecans. The precise synthesis of such architectures has become increasingly feasible through advanced polymerization techniques, particularly ring-opening metathesis polymerization (ROMP). Sequential ROMP strategies enable constructing complex architectures, including triblock bottle-brush copolymers with tailored functionalities and controlled side chain compositions [9,10]. For example, Su et al. demonstrated the synthesis of well-defined triblock bottle-brush polymers using norbornene-based macromonomers, allowing for modular design and post-polymerization modifications that expand their potential in nanomedicine and materials science [10].

Understanding the behavior of bottle-brush polymers in a dilute solution is essential for elucidating their fundamental physicochemical properties. Knowledge of their conformational characteristics is crucial for predicting how these macromolecules behave in more complex biological environments, including their diffusion, interactions with proteins, and stability under physiological conditions [11,12,13]. Moreover, studies of bottle-brush polymers with varying architectures in a dilute solution provide critical benchmarks for theoretical models and simulations, enabling validation of scaling laws and self-consistent field (SCF) theories that describe polymer behavior under different solvent conditions [14].

Coarse-grained computer simulations [15,16,17,18], analytical mean-field theory [19,20,21], and numerical SCF modeling [22,23] consistently indicate that grafting of side chains significantly enhances the stiffness of the main backbone. For large polymerization degree of the side chains the characteristic ratio of the induced persistence length $l_{p}$ to cross-sectional diameter *D* of the bottle-brush (aspect ratio) can reach sufficiently high values ($l_{p}/D\gg 1$), providing a prerequisite for the liquid-crystalline (LC) ordering in semi-dilute solutions of densely grafted molecular brushes [24]. The architecture of the grafts also plays a significant role in determining the characteristic aspect ratio of molecular brushes. Our previous studies [23] have shown that for dendronized polymers, the induced persistence length and effective aspect ratio increase with the degree of branching of the side chains, provided that the translocation of the grafted dendrons is restricted when the main backbone is bent.

While the behavior of molecular brushes with linear side chains is reasonably well understood, the conformational properties of molecular brushes where side chains exhibit comb-like branching remains unexplored. Importantly, such biological supramolecular structures as complexes of aggrecan with hyaluronan can be envisioned as molecular bottle-brushes where grafts (aggrecan monomers) comprise two sequentional domains with different lengths of the secondary side chains (CS and KS, respectively). At the same time, advances in modern synthetic methods now allow for extensive variation in the architecture of the grafts including double-comb (DC) side chains. The DC side chains have two different degrees of polymerization that are arranged in a block-like manner along the backbone. In light of the discussion above, the ability to predict the conformational properties of cylindrical brushes as a function of the architecture of their side chains is a timely and relevant challenge not only for polymer science but also for biomedicine and nanotechnology. The present study aims to provide a theoretical solution to this challenge, with a progressive analysis of the structure-property relationships in cylindrical brushes with DC side chains detailed in the following sections.

## 2. Results and Discussion

### 2.1. Details of Simulations

Cylindrical molecular brushes composed of a primary main chain (backbone) and multiple grafted to it double-comb side chains were modeled on a cubic lattice using the numerical self-consistent field method of Scheutjens-Fleer (see Materials and Methods section) under good (athermal) solvent conditions. Excluded volume interactions between the grafted chains induce elongation and stiffening of the primary backbone of the molecular brush. As a result of these interactions, the backbone is stretched over length scales substantially larger than the cross-sectional brush thickness. Consequently, the molecular brush acquires local cylindrical symmetry, which means that it can undergo only smooth radial bending on scales comparable to or larger than the brush thickness controlled by the size of the side chains, thus justifying a description of the bottle-brush within the framework of cylindrical symmetry. Therefore, to investigate the structural properties of cylindrical brushes formed by comb-like grafts with either uniform or DC architectures, we employed a simplified model, assuming cylindrically symmetric density profile of the monomer units of the comb-like grafts with respect to a straight backbone.

The primary backbone was assumed to be phantom-like, with no excluded-volume interactions with the monomer units of the grafts. This simplification facilitates computational modeling and does not affect the results, provided that the primary backbone is strongly stretched and neighbouring comb-like grafts are strongly overlapped, the conditions fulfilled in the systems studied.

The monomer units comprising the grafted comb-like chains were assumed to be identical in size to each other and to the solvent molecules. The length unit was taken as the linear size of a monomeric unit, *a*, and the energy unit was taken as $k_{B}T$, where $k_{B}$ is the Boltzmann constant, and *T* is the absolute temperature. The grafting density of the primary side chains to the backbone was defined as σ=a/h, where *h* is the distance between the adjacent grafting points of the side chains.

We focused our study on brushes composed of double-comb-like (DC) macromolecules, Figure 11. Each DC macromolecule is attached by one end of its main chain to the primary main chain (backbone) of the bottle-brush.

The secondary side chains in DC polymers were arranged in two domains: adjacent to the backbone (proximal) and peripheral. The secondary side chains in the proximal domain have contour length $n_{1}$ while the side chains in the peripheral domain have length $n_{2}$. The ratio of side-chain lengths in the two domains was adjusted such that the total degree of polymerization of the cylindrical DC molecular brush remained constant

(1) $$N=P_{1}\cdot \left(n_{1}+m_{1}\right)+P_{2}\cdot \left(n_{2}+m_{2}\right)$$

As a particular case, bottle-brushes formed by comb-like grafts with monodisperse secondary side chains, $n_{1}=n_{2}$, were considered. Here, $P_{1}$ and $P_{2}$ denote the numbers of branching points in the proximal (inner) and peripheral (outer) domains of the DC grafts, respectively, and $m_{1}$ and $m_{2}$ are the corresponding contour lengths of the spacers between these branching points. The degree of polymerization of the backbone is determined as

(2) $$N_{b}=P_{1}\cdot m_{1}+P_{2}\cdot m_{2}$$

For simplicity, the cases where $m_{1}=m_{2}=m$ and $P_{1}=P_{2}=P$ were considered.

A comparative analysis of the conformational properties of molecular brushes with monodisperse secondary side chains ($n_{1}=n_{2}=n$) and bidisperse secondary side chains with $n_{1}>n_{2}$ and $n_{2}>n_{1}$ in the grafts was performed. Extreme cases were also examined, in which either the proximal or the peripheral domains represent linear chains (that is, $n_{1}=0$ or $n_{2}=0$).

### 2.2. Molecular Brushes with Comb-like Side Chains

Let us first consider the symmetric case, where the contour lengths of the spacers and secondary side chains are equal in both domains ($n_{1}=n_{2}=n$ and $m_{1}=m_{2}=m$). That is, we consider a cylindrical brush of comb-like chains with side chains of length *n* separated by spacers with length *m*.

An analytical formulation of the self-consistent field theory for planar and curved polymer brushes incorporates a parabolic molecular potential [25,26]:

(3) $$\frac{U(z)}{k_{B}T}=\frac{3\pi^{2}}{8a^{2}}\cdot \frac{\eta^{2}}{N^{2}}\left(H^{2}-z^{2}\right),$$

where *H* is the brush thickness, *a* is the size of the monomer units, *z* is the distance from the grafting surface, $k_{B}$ is the Boltzmann constant, and *T* is the absolute temperature. The parameter η (the topological ratio) characterizes the degree of branching of the brush-forming chains. For comb-shaped grafted chains with long backbones

(4) $$\eta ={\left(1+\frac{n}{m}\right)}^{1/2}.$$

The parabolic shape of the potential in Equation (3) is directly related to the Gaussian (linear) elasticity of the tethered side chains [27]. The volume fraction of monomer units, φ(z), is related to the potential U(z) as:

(5) $$\frac{U(z)}{k_{B}T}=\frac{a^{3}}{k_{B}T}\frac{\delta f_{int}\left[\varphi \left(z\right)\right]}{\delta \varphi (z)}=\frac{\delta }{\delta \varphi (z)}\left[\nu \varphi {\left(z\right)}^{2}+\omega \varphi {\left(z\right)}^{2}+\ldots \right],$$

where $f_{int}\left[\varphi \left(z\right)\right]$ is the free energy density of interactions between monomer units in a semidilute solution, ν=1/2−χ and ω=1/6 are the second and third virial coefficients of the monomer-monomer interactions respectively, χ is Flory-Huggins parameter. Under good solvent conditions (χ<1/2), the polymer volume fraction profile is given by

(6) $$\varphi \left(z\right)=\frac{U(z)}{2\nu k_{B}T}=\frac{3\pi^{2}}{16\nu a^{2}}\cdot \frac{\eta^{2}}{N^{2}}\left(H^{2}-z^{2}\right)$$

Equation (6) is valid in both planar and curved geometries, provided that the potential U(z) supports a finite density of terminal monomer units (free ends) throughout the brush volume. This condition is satisfied in planar and concave brushes, but breaks down in a convex cylindrical brush with formation of the so-called “dead” zone near the brush backbone.

**Figure 1:**
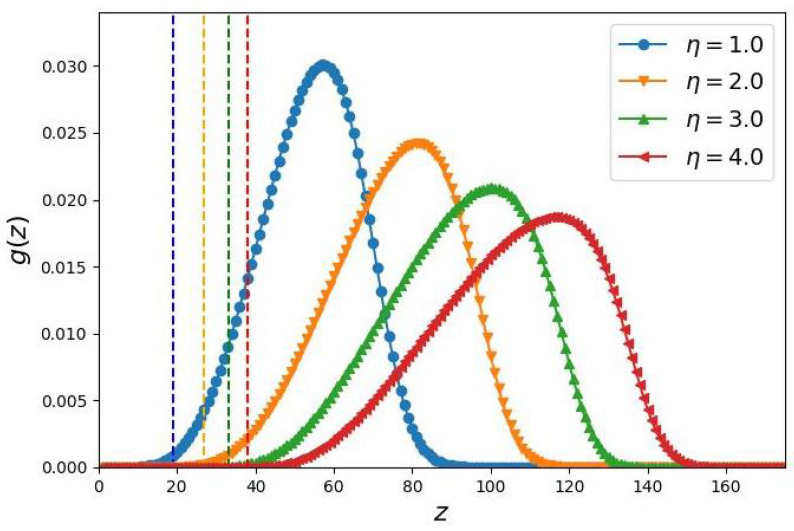
Radial distributions (local volume fractions) of free ends of the main chains of the grafts. All the grafts have the same degree of polymerization $N_{b}=500$ of the main chain, number of monomer units per unit length of the backbone of the bottle-brush Nσ=500, and spacer length m=5 are kept constants as well Branching of the grafts is varied by changing the number of secondary side chains (n=0,15,40,75), corresponding to η=1,2,3,4. Dashed lines indicate the conventional boundaries of the end-free zone (Equation (16)).

The presence of “dead” zones in cylindrical brushes composed of comb-like chains is demonstrated in Figure 1, which shows the results of the numerical SF-SCF simulations. The probability of encountering a terminal segment of the side chain backbone is close to zero in a sufficiently large region of space around the axis of the brush.

The comprehensive account of “dead” zones and the corresponding modification of the parabolic potential in convex polymer brushes of linear polymers was peformed by Ball et al. [28] and Belyi [29] An simplified analytical model accounting for the presence of a “dead” zone in solvated convex brushes of linear polymer chains was developed by Wijmans and Zhulina [30]. Their model combines a presumed power-law decay of the polymer concentration within the “dead” zone with a parabolic profile in the outer brush region. Here, we extend this approach to cylindrical brushes composed of grafted comb-like chains.

The free energy per unit length of the backbone in the “dead” zone is expressed as the sum of the elastic chain-stretching term and the contribution from excluded-volume interactions:

(7) $$F_{d}=AF_{el}+F_{int}=\frac{3A\sigma^{2}\eta^{2}}{2}\int_{0}^{z_{0}}\frac{dz}{\varphi (z)2\pi z}+\nu \int_{0}^{z_{0}}\varphi^{2}\left(z\right)2\pi zdz,$$

Here *A* is a scaling factor that is often introduced in box models to ensure consistency with simulation and experimental data, and $z_{0}$ is the width of the “dead” zone. We have also assumed that within the “dead” zone, only the backbone of the comb-like graft is subject to stretching, while the side chains remain unextended.

Minimizing the free energy $F_{d}$ with respect to φ(z)

(8) $$\frac{\delta F_{d}\left[\varphi \left(z\right)\right]}{\delta \varphi (z)}=0$$

yields the following expression for the polymer volume fraction profile in the “dead” zone:

(9) $$\varphi \left(z\right)={\left[A\frac{3\sigma^{2}\eta^{2}a^{2}}{16\nu \pi^{2}z^{2}}\right]}^{1/3},\;\;\;0<z\leq z_{0},$$

In the outer peripheral zone with distributed free ends of the chains, the volume fraction of monomers remains parabolic:

(10) $$\varphi \left(z\right)=\frac{3\pi^{2}\eta^{2}}{16\nu a^{2}{\left(NX\right)}^{2}}\left[H^{2}-{\left(z-z_{0}\right)}^{2}\right],\;\;\;z_{0}\leq z\leq z_{0}+H$$

with *X* denoting the fraction of monomer units within the peripheral zone, and *H* representing the width of this zone.

The values of *X*, *H*, and $z_{0}$ are specified by three conditions:

(1) continuity of the volume fraction profile φ(z) at $z=z_{0}$:

(11) $${\left[A\frac{3\sigma^{2}\eta^{2}a^{2}}{16\nu \pi^{2}z_{0}^{2}}\right]}^{1/3}=\frac{3\pi^{2}\eta^{2}}{16\nu {\left(NX\right)}^{2}}{\left(\frac{H}{a}\right)}^{2}$$

(2) the normalization condition of φ(z) in the inner layer, ensuring the conservation of a given fraction (1−X) of monomer units:

(12) $$\int_{0}^{z_{0}}\varphi \left(z\right)2\pi zdz=\frac{3}{2}{\left[A\frac{3\pi \sigma^{2}\eta^{2}a^{2}}{16\nu }\right]}^{1/3}z_{0}^{4/3}=N\left(1-X\right)\sigma$$

(3) the normalization condition of φ(z) in the outer layer:

(13) $$\int_{z_{0}}^{z_{0}+H}\varphi \left(z\right)2\pi zdz=\frac{3\pi^{3}\eta^{2}}{32\nu {\left(NX\right)}^{2}}{\left(\frac{H}{a}\right)}^{4}\left(1+\frac{8}{3}\frac{z_{0}}{H}\right)=NX\sigma$$

Solution of the system of Equations (11)–(13) reveals that the ratio of the width of the “dead” zone to that of the peripheral zone yields

(14) $$\frac{z_{0}}{H}=\frac{2\pi }{3\sqrt{A}}\cdot \frac{1-X}{X}$$

Furthermore, the fraction of monomer units *X* in the peripheral zone is a constant value specified as

(15) $$X=\frac{12\pi^{2}-16\pi \sqrt{A}}{9A-16\pi \sqrt{A}+12\pi^{2}}$$

**Figure 2:**
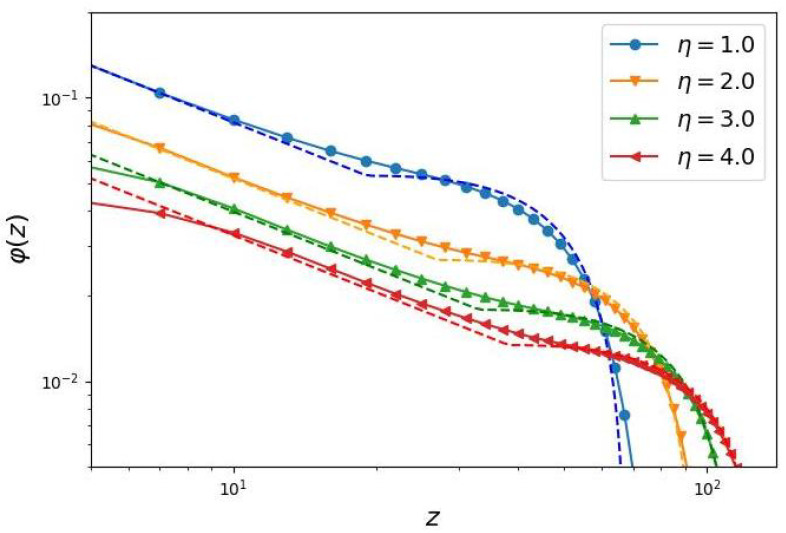
Radial profiles of the volume fraction distribution of the side chain monomer units in log-log coordinates. The system parameters correspond to the previous graph. The dashed lines show the analytically obtained profiles. The same color scheme is used for the curves as in Figure 1, representing identical brush parameters.

The approximation of the numerically modeled profiles φ(z) using an analytical function (Equation (9)) results in a best-fit parameter value of A=1.45 (see Figure 2). At A=1.45, this fraction *X* is equal to 0.816, indicating that, regardless of the grafting density, the degree of polymerization or architecture of the grafted comb-like chains, approximately 82% of the monomer units reside in the peripheral zone, while only about 18% are located in the “dead” zone. The width of the “dead” zone is approximately three times smaller than the width of the peripheral zone (the ratio $z_{0}/H=0.39$).

The width $z_{0}$ of the “dead” zone is described by the following equation:

(16) $$\frac{z_{0}}{a}=\frac{2}{3}{\left(\frac{8\nu }{\pi A}\right)}^{1/4}{\left(1-X\right)}^{3/4}N^{3/4}\sigma^{1/4}\eta^{-1/2}$$

while the width *H* differs from $z_{0}$ in Equation (16) only by the numerical prefactor:

(17) $$\frac{H}{a}={\left(\frac{32\nu }{3\pi^{3}}\right)}^{1/4}{\left(1+\frac{8z_{0}}{3H}\right)}^{-1/4}X^{3/4}N^{3/4}\sigma^{1/4}\eta^{-1/2}$$

The derived formulas (Equations (15)–(17)) allow for the construction of the analytical volume fraction profiles (9) and (10), taking into account “dead” zones. A comparison between the proposed analytical theory and direct numerical simulations is shown in Figure 2. The results demonstrate reasonably good agreement.

The average half-thickness of a molecular brush can be characterized by the first moment of the radial distribution of the polymer volume fraction around the brush axis:

(18) $$\langle H\rangle =\frac{\int_{0}^{z_{0}+H}\varphi \left(z\right)2\pi z^{2}dz}{\int_{0}^{z_{0}+H}\varphi \left(z\right)2\pi zdz}$$

or alternatively, by the first moment of the radial distribution of the free ends of the grafted comb-like side chains

(19) $$\langle H_{e}\rangle =\frac{\int_{0}^{z_{0}+H}g\left(z\right)2\pi z^{2}dz}{\int_{0}^{z_{0}+H}g\left(z\right)2\pi zdz}.$$

**Figure 3:**
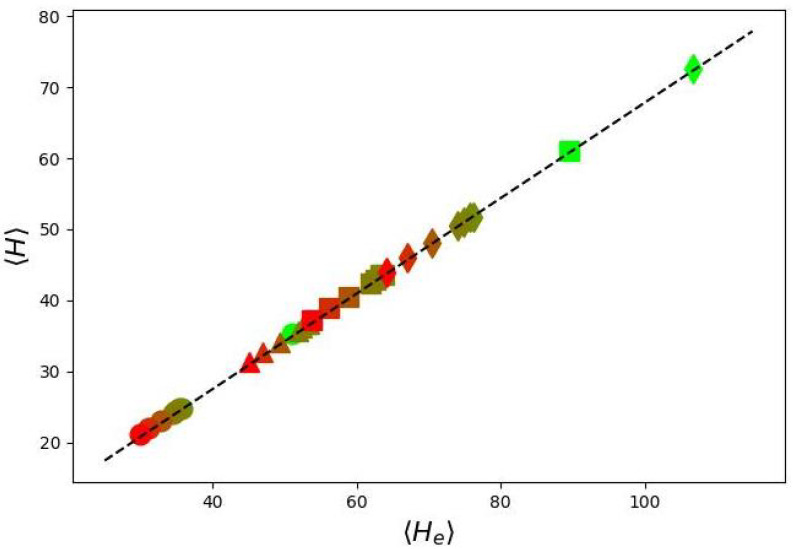
Dependence of the first moment of the distribution of the volume fraction profile of monomer units on the first moment of the distribution of the free ends of the main chain of grafted chains. The data points represent the results of numerical simulations, and the dashed lines show the approximate dependence $\langle H\rangle =1.68\langle H_{e}\rangle -15$. Different symbols correspond to varied grating density σ= 0.1 (circles); 0.5 (triangles); 1.0 (squares); 2.0 (diamonds). The color of the symbols gradually changes from red to green upon an increase in branching degree η from 1 to 3. The polymerization degree of the side chains is N=1000.

As shown in Figure 3, for cylindrical molecular brushes composed of monodisperse comb-like side chains under good solvent conditions, these two characteristics are directly proportional.

If the “dead” zone is neglected and the parabolic potential is extended to the whole cylindrical brush, the first moment 〈H〉 takes the following form:

(20) $$\langle H\rangle =\frac{8}{15}H=\frac{8a}{15}{\left(\frac{32\nu }{3\pi^{3}}\right)}^{1/4}N^{3/4}\sigma^{1/4}\eta^{-1/2}$$

**Figure 4:**
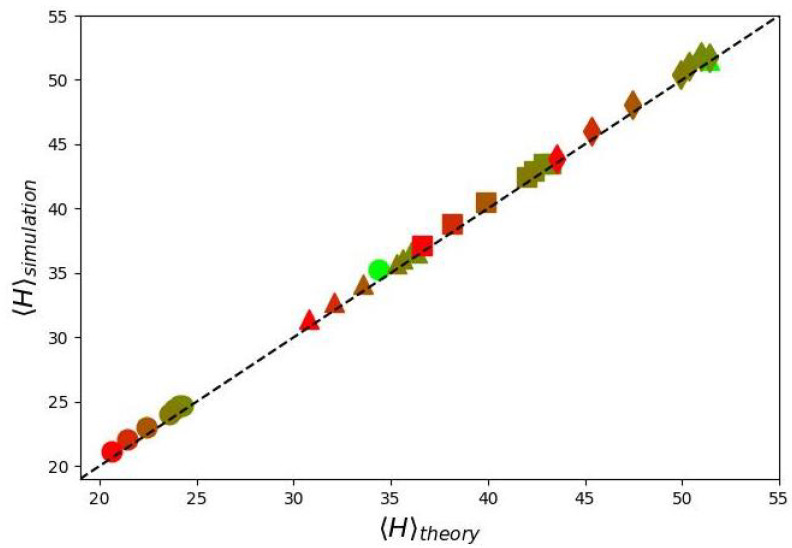
Dependence of the first moment of the radial monomer density distribution obtained from numerical simulations ${\langle H\rangle }_{simulation}$ on the corresponding analytically predicted value ${\langle H\rangle }_{theory}$. The color scheme matches that used in the previous plots.

Surprisingly, despite of a significant effect of “dead” zone on the shape of φ(z), Equation (20) provides a reasonably accurate description of the numerically calculated 〈H〉 (see Figure 4).

### 2.3. Molecular Bruhes with Double-Comb (DC) Side Chains

We now consider cylindrical molecular brushes composed of double-comb (DC) chains. In all cases, the total number of secondary side chains per DC is kept constant at 20, and the secondary side chains are grafted onto every third monomeric unit along the main chains of the DCs (m=3). The degrees of polymerization of the secondary side chains in proximal and peripheral domains of the bottle-brush forming DCs ( ($n_{1}$ and $n_{2}$, respectively) are varied so that $n_{1}+n_{2}=50$, ensuring a constant total degree of polymerization of the DCs (N=1124). The branching parameter $\eta_{i}={\left(1+n_{i}/m\right)}^{1/2}$ (i=1,2), is tuned solely by adjusting the contour length $n_{i}$ of the side chain in each domain.

**Figure 5:**
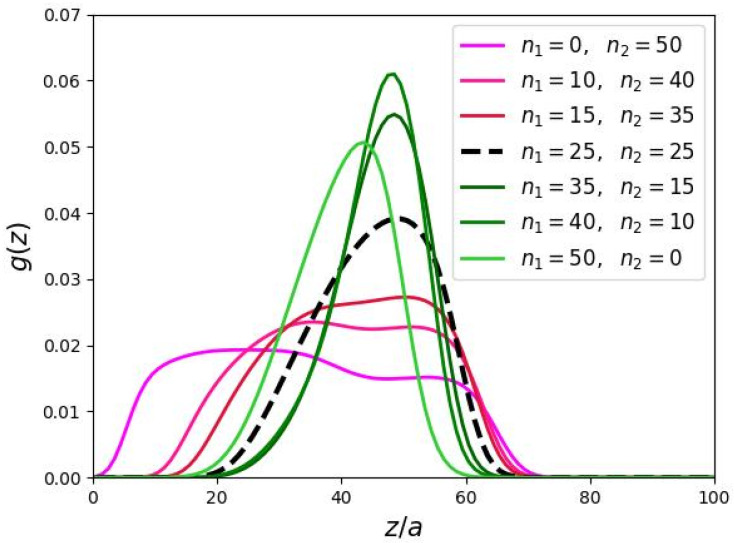
Radial distribution of the end segments of the backbones of DCs in cylindrical molecular brushes (a/h=0.5,N=1124,m=3).

The radial distributions g(z) of the end-points of the DC backbones are shown in Figure 5. When branching is increased in the peripheral domain, the width of the “dead” zone decreases, indicating a more uniform spatial distribution of end-points across the brush volume. In contrast, increasing branching in the proximal domain leads to reduced stretching of the DCs main chains, but their end-point distributions remain sharp.

**Figure 6:**
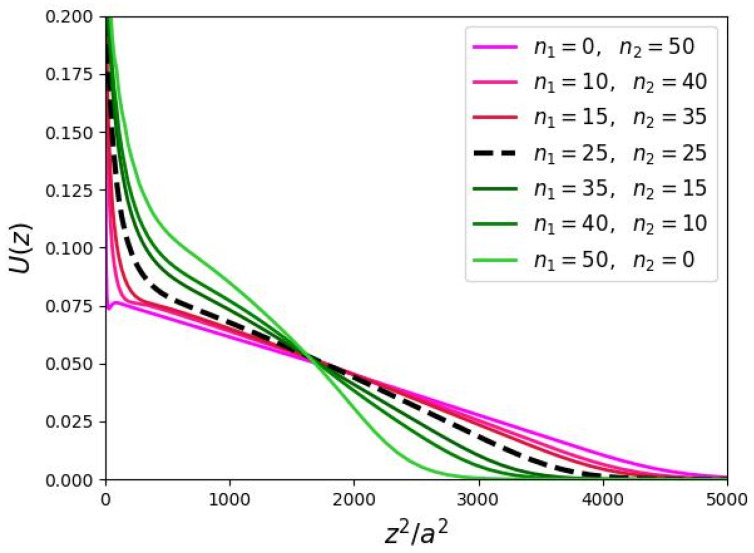
The molecular potential in the bottle-brush of DC chains as a function of $z^{2}$, with *z* measured from the brush backbone. (a/h=0.5,N=1124,m=3).

As shown in the previous section, the analytical theory predicts a parabolic shape of the molecular potential (a linear dependence of the potential on the squared radial distance $z^{2}$ from the brush axis) for bottle-brushes formed by comb-like polymers with monodisperse side chains ($n_{1}=n_{2}$) in the major part of the brush. However, an increase in length $n_{1}$ of the side chain in the proximal domain combined with the decrease in length $n_{2}$ to the side chain in the peripheral domain, leads to significant deviations in U(z) from the parabolic shape (Figure 6).

**Figure 7:**
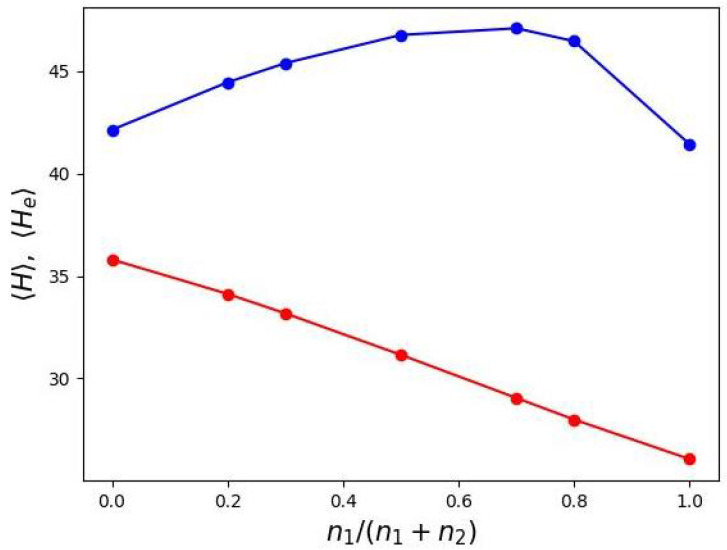
The first moments 〈H〉 (red line) and $\langle H_{e}\rangle$ (blue line) are plotted as a function of the relative contour lengths of the secondary side chain in the proximal domain of the DC macromolecule.

The non-monotonic variation of the “dead” zone width with ratio $n_{1}/\left(n_{1}+n_{2}\right)$ is reflected in the behavior of the first moments 〈H〉 and $\langle H_{e}\rangle$ (Equations (18) and (19)). In contrast to the monodisperse case ($n_{1}=n_{2}$) in which 〈H〉 and $\langle H_{e}\rangle$ are proportional, their proportionality breaks down for DC grafts. While 〈H〉 decreases monotonically and nearly linearly with increasing $n_{1}/\left(n_{1}+n_{2}\right)$, $\langle H_{e}\rangle$ exhibits a pronounced maximum as a function of $n_{1}/\left(n_{1}+n_{2}\right)$ (Figure 7).

To evaluate how the architecture of the grafted DC polymers influences tension imposed on the brush axis, the free energy *F* per grafted chain was calculated as a function of distance *h* between grafting points. In the parabolic potential framework, the free energy per chain in a cylindricaal brush with monodisperse comb-like grafts is given by:

(21) $$F=\frac{3\pi^{5}\eta^{4}}{128\nu N^{4}}\cdot \frac{h}{a}\cdot {\left(\frac{H}{a}\right)}^{6}=\frac{3\pi^{1/2}\nu^{1/2}}{128}{\left(\frac{32\nu }{3}\right)}^{3/2}\cdot {\left(\frac{h}{a}\right)}^{-1/2}\eta N^{1/2}$$

and axial force is then obtained as the negative derivative of this free energy with respect to *h*:

(22) $$f\left(h\right)=-\frac{dF(h)}{dh}=\frac{3\pi^{1/2}\nu^{1/2}}{256}{\left(\frac{32\nu }{3}\right)}^{3/2}\cdot {\left(\frac{h}{a}\right)}^{-3/2}\eta N^{1/2}$$

As it follows from Equation (22), axial tension *f* imposed by the grafts in the brush backbone is expected to increase upon increasing branching of the grafts (increase in η) and decrease as $h^{-3/2}$ as a function of distance *h* between grafts.

**Figure 8:**
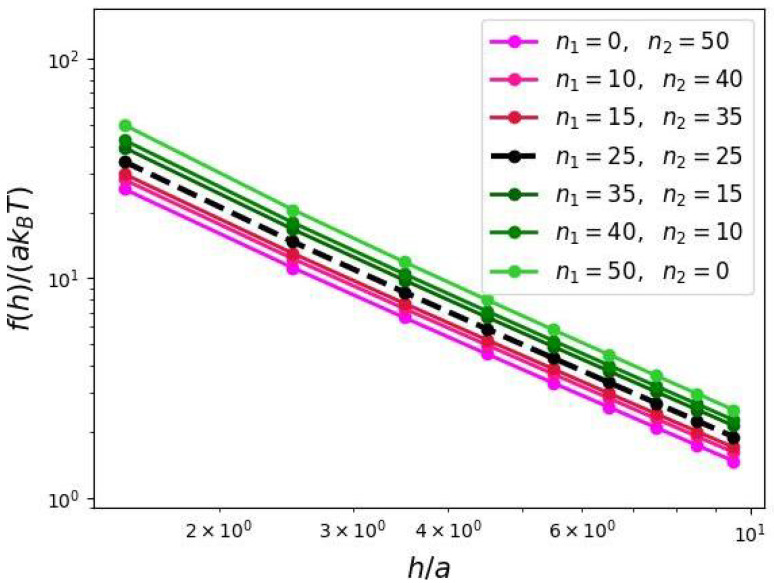
Dependence of the axial force stretching the backbone of the molecular brush due to repulsions of comb-like grafts (force values are normalized per grafted chain; the slope in double-logarithmic coordinates is −3/2).

The results of SF-SCF calculations presented in Figure 8 indicate that DC chains with proximal domain containing a larger fraction of the total polymer material exhibit a stronger tension on the brush axis compared to those with a dominant peripheral domain. In all cases the numerically calculated dependences f(h) demonstrate the value of exponent close to −3/2 predicted by Equation (22).

**Figure 9:**
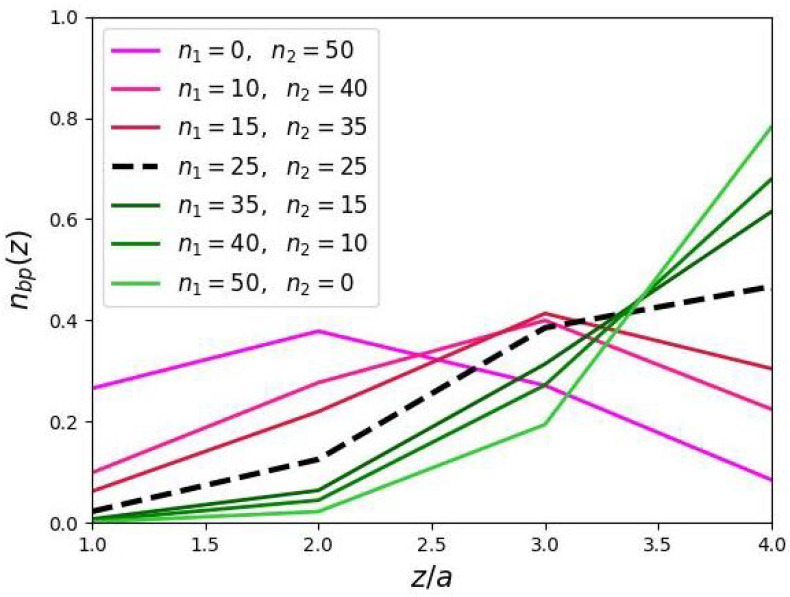
The probability of finding the first branching point in a grafted macromolecule at a distance *z* from the backbone.

To minimize steric interactions, the side chains tend to extend in the radial direction, exerting a “detatching” force at the grafting point perpendicular to the backbone. This effect is best illustrated by the stretching of the root spacer, i.e., the spacer between the grafting point and the first branching point in the grafted DC chain (Figure 9). As shown in Figure 9, the elongation of the root spacer correlates with the axial force exerted on the brush axis by DC chains with more massive peripheral domain (mimicing aggrecan monomers). The greater the axial force, the greater the stretching of the root spacer.

To assess the contribution of side chain interactions to the enhanced bending rigidity of a bottle-brush polymer, a uniform curvature is applied to the backbone, characterized by a radius *R* much larger than the brush half-thickness *H*. The resulting excess free energy per grafted side chain, induced by bending at radius *R*, can be determined through the following expansion:

(23) $$\Delta F\left(R\right)=F\left(1/R=0\right)\left[a_{2}{\left(\frac{H}{R}\right)}^{2}+a_{4}{\left(\frac{H}{R}\right)}^{4}+\ldots \right]$$

Due to symmetry, the odd-order terms in the expansion vanish. The leading contributions arise from the even-order terms, where $a_{2}$ and $a_{4}$ are dimensionless numerical coefficients. By definition, the bending modulus $\kappa_{p}$ characterizes the free energy cost of bending,

(24) $$\frac{\Delta F(R)}{h}=\frac{\kappa_{p}}{2R}+\ldots$$

The induced persistence length $l_{p}$ is related to the bending modulus $\kappa_{p}$ as

(25) $$l_{p}=\frac{\kappa_{p}}{k_{B}T}∼\frac{FH^{2}}{hk_{B}T}$$

Based on the above equations, as well as Equations (17) and (21), it is straightforward to show that under athermal solvent conditions the induced persistence length is equal to

(26) $$\frac{l_{p}}{a}=0.046{\left(\frac{Na}{h}\right)}^{2}$$

and does not depend on the architecture of the grafted chains given their identical grafting density and degree of polymerization.

**Figure 10:**
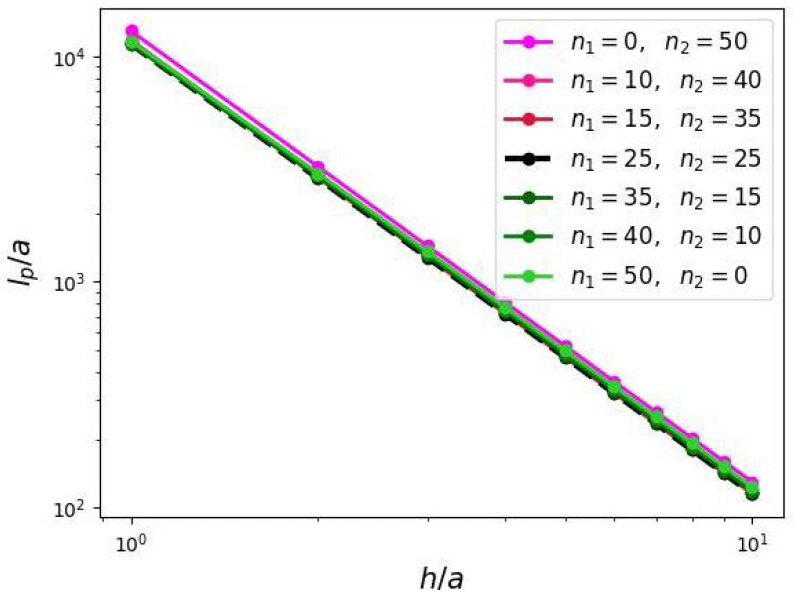
Induced persistence length due to grafting of comb-like side chains (slope of −2 in double-logarithmic coordinates).

The applied here numerical SF-SCF method allows for direct calculation of the free energy *F* and the brush thickness *H* in the brush straight configuration. Then the induced persistent length $l_{p}$ can be evaluated using Equation (25). The results of SF-SCF numerical evaluation of $l_{p}\left(h\right)$ are presented in Figure 10. As the distance *h* between grafting points of the side chains along the brush axis increases, the induced persistence length $l_{p}$ decreases. These dependences follow power-law behavior with exponents of −2, in agreement with Equation (26), regardless of the distribution of monomer units of secondary side chains between proximal and peripheral domains of the bottle-brush-forming DC chains.

The performed SF-SCF modeling demonstrated that molecular brushes composed of DC side chains exhibit a fundamentally different structure compared to bottle-brushes with monodisperse comb-shaped chains. By tuning the ratio of lengths of the side chains in proximal and peripheral domains, the spatial distribution of the end-points of the main chains of the grafts within the brush could be precisely controlled. The latter could be a key factor in designing molecular-brush-based nanocontainers for targeted substance delivery. Furthermore, adjusting the architecture of grafted DC chains during synthesis allows for a significant modulation of the brush thickness while preserving intact the induced backbone rigidity. This feature enables regulation of the liquid-crystalline behavior in semi-dilute solutions and the mechanical properties of bottle-brush-based gels.

## 3. Materials and Methods

**Figure 11:**
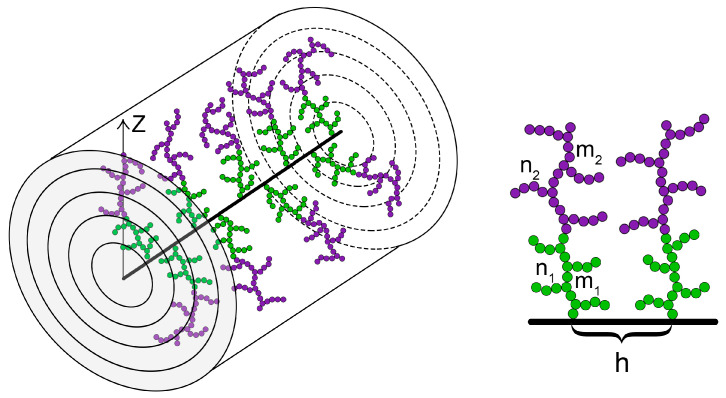
Schematic representation of a molecular brush with DC side chains composed of two domains: a proximal domain (green) and a peripheral domain (violet).

The numerical approach SF-SCF of Scheutjens and Fleer [31] is, like the analytical method, based on the mean-field approximation. This approach employs a discrete lattice representation of the space. The system is modeled as a cubic lattice with a spacing equal to the linear size of a monomer unit *a*. The lattice sites are organized into layers arranged in cylindrical shells around the brush backbone and labeled $z=0,\ldots ,z_{max}$. The layer z=0 corresponds to the backbone, and the first segment of each grafted chain is anchored in the layer z=1. The volume fraction of the monomer units φ and the potential *u* are assumed to be uniform within each layer and vary only in the radial direction *z* (see Figure 11).

The main and side chains of the grafted macromolecules are treated as freely joined chains in an external effective field u(z), induced by intermolecular and intramolecular interactions. Correlations of longer range are neglected. The overlap of two segments at a lattice site is allowed but strongly suppressed by the incompressibility condition:

(27) $$\varphi \left(z\right)+\varphi_{s}\left(z\right)=1,\;\;\forall z,$$

where φ(z) and $\varphi_{s}\left(z\right)$ are the volume fractions of polymer and solvent, respectively.

The free energy per macromolecule, in the case of an athermal solvent, is defined as the negative logarithm of the partition function of the grafted macromolecules in the effective potential field (−lnQ[u]), minus the work done by the field, $\sum_{z}u\left(z\right)\varphi \left(z\right)$:

(28) $$F=-\ln Q\left[u\left(z\right)\right]-\frac{1}{\sigma }\sum_{z=0}^{z=z_{max}}u\left(z\right)\varphi \left(z\right)L\left(z\right),$$

where $L\left(z\right)=A\left(z\right)/a^{2}$ is the dimensionless cross-sectional area of the *z*-th cylindrical layer.

Calculating the volume fraction profile φ(z) and the potential profile u(z), corresponding to the minimum free energy under the incompressibility constraint, is achieved by minimizing the free energy functional:

(29) $$F=F\left\{\varphi \left(z\right),u\left(z\right)\right\}+\sum_{z=0}^{z=D}\alpha \left(z\right)\left[\varphi \left(z\right)+\varphi_{s}\left(z\right)-1\right],$$

where α(z) the Lagrange multiplier field (a set of Lagrange multipliers).

Minimizing the functional F with respect to φ(z) yields the expression for the potential u(z):

(30) $$\frac{\delta F}{\delta \varphi (z)}=0\;\;\;\Rightarrow \;\;\;u\left(z\right)\leftarrow \alpha \left(z\right)$$

Minimization of F with respect to u(z) provides a way to compute the volume fraction distribution φ(z):

(31) $$\frac{\delta F}{\delta u(z)}=0\;\;\;\Rightarrow \;\;\;\varphi \left(z\right)\leftarrow \frac{\delta (-\ln Q)}{\delta u(z)}$$

Here, the partition function *Q* is computed using specialized propagator matrices.

The free energy minimization is performed iteratively. At each iteration, new values of the polymer volume fraction are computed from the current potential values, and new potential values are calculated from the current volume fraction. The procedure continues until self-consistency is achieved to a specified tolerance.

At the initial step, an arbitrary Lagrange multiplier field α(z) is initialized. Then, α(z) is updated iteratively using a gradient descent procedure:

(32) $$\alpha \left(z\right)\leftarrow \alpha +h\frac{\delta F}{\delta \alpha (z)}=\alpha \left(z\right)+h\left[\varphi_{br}\left(z\right)+\varphi_{lp}\left(z\right)-1\right],$$

where h<1/2—is the size of the convergence step.

The overall computational algorithm is described in detail in the original reference on the method [31].

## 4. Conclusions

In this work, we present a combined numerical (SF-SCF) and analytical theoretical study of cylindrical molecular brushes formed by double-comb (DC) polymers tethered to a rigid linear backbone. The SF-SCF modeling demonstrates that molecular brushes composed of DC polymers with different lengths of secondary side chains in proximal to the grafting point and peripheral domains exhibit a fundamentally different conformational behavior compared to those with monodisperse secondary side chains. In molecular brushes formed by comb-shaped macromolecules with uniform secondary side chain length, the radial distribution g(z) of main chain ends is unimodal, and the cross-sectional thickness *H* of the bottle-brush is directly correlated with the topological ratio, η. At fixed number of monomer units in the grafts per unit length of the bottle-brush axis the width of “dead” zone monotonously increases with topological ratio η.

In contrast, molecular brushes with DC side chains are characterized by asymmetric distribution of monomer units between the proximal (adjacent to the brush axis) and peripheral (terminal) domains, that leads to a more complex conformational landscape. Spatial distribution of the main chain ends within the brush can be controlled by tuning the ratio of lengths of the side chains in the proximal and peripheral domains, resulting in either sharp unimodal or smooth weakly bimodal end-point distributions. Notably, increasing the length of side chains in the peripheral domain reduces the extent of the “dead” zone and promotes a more uniform occupancy of the brush volume, whereas enhancing the side chain length in the proximal domain leads to a denser inner region and increasing “dead” zone width. Despite these significant architectural differences, the induced persistence length of molecular brush with DC side chains remains fairly constant for fixed grafting density and total degree of polymerization of the grafts. This reveals a fundamental decoupling between local conformational structure and thermodynamic rigidity: while the thickness of the brush and the spatial distribution of the free ends can be precisely tuned by adjusting the architecture of the grafts, the large-scale stiffness of the backbone is mainly determined by overall grafting density and total degree of polymerization of the side chain.

This unique feature enables independent tuning of structural and mechanical properties, offering new opportunities for the design of advanced bottle-brush-based biomimetic materials, such as nanocontainers for targeted drug delivery, responsive hydrogels, and systems with controllable liquid-crystalline ordering in semi-dilute solutions.

Finally, our findings provide a rational explanation for evolutionarily-selected inhomogenuous distribution of GAG chains mass along the contour of the aggrecan monomers: enrichment of the domain of the aggrecan proximal to the junction with HA by shorter KS side chains allows for reducing the normal (tearing) force and thus for enhancing the aggrecan-HA complex stability while maintaining its high hydration degree (swelling capability).

## Data Availability

The original contributions presented in this study are included in the article. Further inquiries can be directed to the corresponding author.

## Abbreviations

The following abbreviations are used in this manuscript:

GAG	Glycosaminoglycan
ROMP	Ring-opening metathesis polymerization
LC (ordering)	The liquid-crystalline (ordering)
SCF (method)	The self-consistent field (method)
